# A comparison of methods for automated motion correction of DCE-MRI perfusion datasets evaluated in terms of diagnostic accuracy: a CE-MARC sub-study

**DOI:** 10.1186/1532-429X-16-S1-P206

**Published:** 2014-01-16

**Authors:** Constantine Zakkaroff, Aleksandra Radjenovic, John D Biglands, Sven Plein, John P Greenwood, Derek R Magee

**Affiliations:** 1 Division of Medical Physics, University of Leeds, Leeds, UK; 2 School of Computing, University of Leeds, Leeds, UK; 3 MCRC & LIGHT, University of Leeds, Leeds, UK; 4 Institute of Cardiovascular and Medical Sciences, BHF Glasgow Cardiovascular Centre, University of Glasgow, Leeds, UK

## Background

Automated mage registration in cardiac myocardial perfusion is a necessity before quantitative perfusion can be widely accepted in clinical practice. Increasingly complex motion correction algorithms are being developed to deal with cardiac motion. However, the impact of these improvements has not been evaluated in terms of the final clinical diagnosis. Advanced motion correction methods are associated with increased computational overhead and the potential of introducing subtle registration errors, which can be hard to detect and quantify. The aim of this study was to compare the performance of the various automated correction methods in terms of their impact on diagnostic accuracy.

## Methods

**Figure 1:**
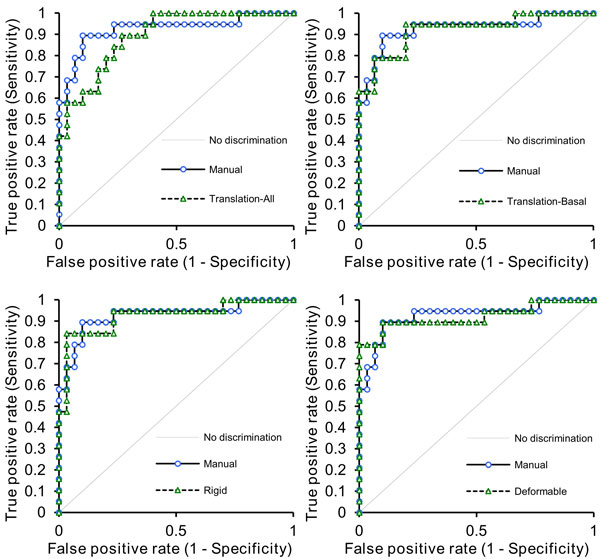
**ROC curves generated for all four methods of motion correction**.

This was a retrospective sub-study using data from the CE-MARC trial (Greenwood et al., Lancet, 2012). A 50-patient sample was selected such that the distribution of risk factors and disease status within the sample was representative of the full CE-MARC cohort. Three strategies for motion correction with the mutual information image similarity metric were used: 1) independent translation correction for all slices, 2) translation correction for the basal slice with transform propagation to the remaining two slices, assuming identical motion for these slices, 3) rigid correction (translation and rotation) for the basal slice with transform propagation as in (2) and 4) deformable correction in all slices. Contours depicting the myocardium and a region within the left blood pool were drawn on the dynamic frame exhibiting the best visual contrast on the three slices. These contours were propagated to the remaining frames (and slices) of the dataset using each motion correction strategy. Quantitative myocardial blood flow (MBF) estimates were obtained using Fermi-constrained deconvolution and myocardial perfusion reserve (MPR) indices were calculated from the ratio of stress to rest MBF estimates. The presence of myocardial ischaemia was assessed using the consensus diagnosis of invasive, quantitative X-ray angiography and myocardial Single Photon Computed Tomography (SPECT) imaging. Receiver Operator Characteristic (ROC) curves were generated for each motion correction strategy (Figure 1). A DeLong, DeLong, Clarke-Pearson comparison was used to test for statistically significant differences in the area under the curve (AUC) values of each strategy in comparison to the MPR indices for the manually corrected series.

## Results

There were no significant differences between the diagnostic accuracy of the four automatic correction strategies (Table 1).

**Table 1:** AUC and p values comparing automated motion correction and manual motion correction (AUC = 0.93) for all four methods.

Translation (All slices)	Translation (Basal slice)	Rigid (Basal slice)	Deformable (All slices)
0.89 (p = 0.41)	0.92 (p = 0.88)	0.93 (p = 1.00)	0.92 (p = 0.91)

## Conclusions

We have shown that the simplest automated motion correction method (method 2 with translation transform for basal location and subsequent transform propagation) provides a diagnostic accuracy equivalent to the progressively more complex methods of motion correction.

## Funding

This work was funded by the Top Achiever Doctoral scholarship awarded by the Tertiary Education Commission of New Zealand.

